# 1854. Legionnaires’ Disease Case Exposure Classification Differs by Race and Ethnicity and Neighborhood Health — California, 2011–2021

**DOI:** 10.1093/ofid/ofad500.1682

**Published:** 2023-11-27

**Authors:** Cassandra O Schember, Sarah Rutschmann, Akiko Kimura, Alyssa Nguyen, Duc Vugia, Seema Jain

**Affiliations:** Centers for Disease Cntrol and Prevention, Emeryville, California; California Department of Public Health, Richmond, California; CDPH, Los Angeles, California; California Department of Public Health, Richmond, California; California Department of Public Health, Richmond, California; California Department of Public Health, Richmond, California

## Abstract

**Background:**

Legionnaires’ disease (LD) is a severe pneumonia caused by *Legionella* bacteria transmitted by inhalation of contaminated water droplets found in poorly maintained water systems. During 2012–2020, 53% of California LD patients were white and 14% were black; however, these groups account for only 39% and 6% of the population, respectively. Healthy Places Index (HPI) measures census tract health, which can affect risk for LD, by combining 23 indicators of social and environmental conditions. We assessed associations between race/ethnicity and neighborhood health, with LD case exposure classifications to establish priorities for decreasing LD disparities in California.

**Methods:**

Using all California LD cases reported during 2011–2021 and six, individual modified Poisson regression models, we estimated incidence rate ratios (IRRs) and 95% confidence intervals (CIs) for associations between race/ethnicity and neighborhood health (using HPI quartile) with three LD case exposure classifications (sporadic, healthcare-associated, or travel-associated). The most advantaged HPI quartile and non-Hispanic white race/ethnicity were the referent groups. All models controlled for birth sex, age, and year.

**Results:**

Among 4,373 people with LD, 47% were white, 22% were Hispanic, 12% were black, and 22%–28% fell in each HPI quartile. Among cases, 78% were sporadic, 16% were travel-associated, and 5% were healthcare-associated. Black (IRR: 0.69; 95% CI: 0.55, 0.87) and Hispanic (IRR: 0.64; 95% CI: 0.52, 0.77) race/ethnicity were associated with decreased rates of travel-associated LD. Black (IRR: 1.06; 95% CI: 1.01, 1.11) and Hispanic (IRR: 1.11; 95% CI: 1.07, 1.16) race/ethnicity, and living in the least advantaged quartile (IRR: 1.05; 95% CI: 1.01, 1.10) were associated with increased rates of sporadic LD. Healthcare-associated LD was not associated with race/ethnicity or HPI.

**Conclusion:**

We found evidence of racial and neighborhood disparities in sporadic LD, racial disparities in travel-associated LD, and no evidence of disparities in healthcare-associated LD. Efforts to increase uptake of water management programs in buildings in disadvantaged neighborhoods and travel accommodations might lessen LD burden and disparities in California.

**Disclosures:**

**All Authors**: No reported disclosures

